# Knowledge of non-communicable diseases among adolescents in Uasin Gishu County, Kenya

**DOI:** 10.4314/ahs.v23i2.68

**Published:** 2023-06

**Authors:** Sharon Jemutai Kiplagat, Tania Steyl, Lucy-Joy Wachira, Joliana Phillips

**Affiliations:** 1 Department of Physiotherapy, University of the Western Cape, Cape Town, South Africa; 2 Department of Orthopaedics and Rehabilitation, Moi University, Eldoret, Kenya; 3 Department of Physical Education, Exercise and Sports Science, Kenyatta University, Nairobi, Kenya

**Keywords:** Non-communicable diseases, risk factors, adolescents, knowledge, Kenya

## Abstract

**Introduction:**

Exposure to risk factors of non-communicable diseases (NCDs) begins early especially during adolescence while morbidity and mortality occurs mainly in adulthood.

**Objective:**

To assess the level of knowledge of major NCDs (cardiovascular disease, cancer, and diabetes) and risk factors among adolescents in a semi-urban setting.

**Methods:**

A descriptive cross-sectional survey was used to collect data. The study targeted students attending mixed secondary schools in Uasin Gishu County in Kenya. An adapted knowledge assessment questionnaire relating to chronic diseases of lifestyle was used for data collection. Data was analysed using SPSS version 25.

**Results:**

A total of 1,281 students participated in the study. The results revealed that most of the participants (62.1%) had low levels of knowledge about NCD risk factors. Very few students in the present study recognized the role of family history and genetic predisposition as potential risk factors of hypertension (18%) and diabetes (24.7%) as well as the normal blood pressure (35.9%) and blood glucose levels (20.5%). Although most of the students identified alcohol and smoking as risk factors for cancer, half (51.6%) of them did not have the knowledge that regular physical activity reduces the risk of getting cancer.

**Conclusion:**

Many of the adolescents in Uasin Gishu County, Kenya had low level of knowledge regarding NCDs and their risk factors. The findings of this study highlight the need for a school-based intervention programme to raise awareness of non-communicable disease risk factors among adolescents.

## Introduction

Non-communicable diseases (NCDs) also known as chronic diseases have become a major public health problem worldwide. Major NCDs include cardiovascular diseases, diabetes, cancer and respiratory diseases which constitute 80% of all premature NCD deaths according to World Health Organization^1^. Among the main contributing factors are physical inactivity, unhealthy diet, harmful use of alcohol and tobacco^1^. NCD risk factors have been reported among adolescents and youth globally. An African study among senior high school students reported high levels of physical inactivity (75%), overweight or obesity (15%), alcohol consumption (7.0%) and tobacco use 2.5% 2. Another study conducted among high school students in an urban setting in India indicated a high prevalence in NCD risk factors such as cigarette smoking exposure (75.5%), lack of physical activity (21.8%) and taking carbonated drinks more than three times per week (30.2%) 3. A report from over 100 countries in Europe on health behaviours distribution among adolescents found that approximately 80% of them performed daily physical activities (for at least 60 min), 32% used the computer for >2 hours per day, 6% smoked cigarettes daily, 7.6% consumed beer weekly, and 25% had an unhealthy diet^4^. In addition, more than a quarter (26.5%) 15–19-year-olds amounting to 155 million adolescents are current drinkers^5^.

Over the past decade, evidence from several local studies revealed that Kenyan youth engage in substance abuse^6^, ^7^, ^8^, have unhealthy dietary habits^9^ and although majority have favourable physical activity (PA) levels^10^ very few (12.6%) met the recommended PA threshold of at least 60 min per day^11^. A study conducted in the same setting (Eldoret town) as this study revealed that the mean daily time spent in moderate to vigorous physical activity (MVPA) among children was 44 minutes, with 16.5% of them meeting physical activity guidelines^12^.

NCD risk factors have an impact on the future life and health of the adolescents. For instance, studies have found that adolescents who are overweight are twice more likely to develop cardiovascular diseases (CVD) and have seven times greater risk of atherosclerosis when they become adults^13^. Furthermore, almost three out of four obese adolescents remain obese when they become adults, and these increases their risk of getting heart disease, stroke, type 2 diabetes and cancers. It has also been noted that adolescents who begun drinking alcohol before the age of 15 are five times more likely to abuse alcohol in adulthood compared to those who begin drinking at 19 years or older^14^. In addition, five percent of all deaths of young people aged between 15 and 29 is attributed to alcohol use^5^.

A study on nutrition knowledge, attitude and practices among primary school children in Nairobi, Kenya reported that half of the pupils had moderate knowledge of nutrition and poor dietary practices which were associated with negative attitude towards diet^9^. Evidence of adolescents NCDs current knowledge and lifestyle-related practices would be of help to both the health and education sectors with planning and implementation of much needed programs for adolescents. Limited published studies are available on knowledge of common NCDs among secondary school students in Kenya. Hence, it is essential to assess the knowledge of secondary school students concerning NCDs and their risk factors in order to guide the development of an intervention programme. The aim of this study, therefore, was to determine the level of knowledge of common NCDs (diabetes, hypertension and cancer) and their risk factors among secondary school students in Uasin Gishu County, Kenya.

## Methods

### Study design and setting

A cross-sectional descriptive survey design was employed for this study. Uasin Gishu is located in the former Rift Valley province. The county has the second highest population in the province with a population of 1,163,186 as per the 2019 population census. Uasin Gishu County is metropolitan with a mix of urban and rural settings and is home to a diverse population with varying backgrounds, socio-cultural and socioeconomic characteristics. The schools in Uasin Gishu County are categorized into Eldoret East, Wareng and Eldoret West sub-counties with 45, 53 and 72 public secondary schools respectively.

### Sampling

The schools were selected using stratified proportional sampling from a list of all schools obtained from the county education office as per the year 2018 leading to a total of 16 mixed day/boarding secondary schools. The three sub-counties formed the strata and every 10^th^ school was picked. Only ten schools agreed to participate in the study (four schools had both day scholars and boarders while the remaining 6 schools had day scholars only). The inclusion criteria were students in forms one to four also known as grade 9 to grade 12 (this are all the year groups in the secondary school system in Kenya) from mixed day/boarding secondary schools in Uasin Gishu County. Mixed schools were preferred over single gender schools since it has the largest student population. In addition, both genders co-exist and experience similar predisposing factors, behavioural influence and environmental conditions. Students without written informed consent and assent forms were excluded from the study.

Yamane formula n=N/ 1+N (e)^2^ was used to calculate the student sample in each school. The representative sample size of students was 1,310. Within each class, simple random sampling was used to select the participants based on the student registration numbers obtained from the institutions' heads.

### Ethical considerations

Ethics approval was obtained from the Biomedical Research Ethics Committee at the University of the Western Cape (BM18/1/1) and the Institutional Research and Ethics Committee (IREC), of Moi University and Moi Teaching and Referral Hospital (IREC/2017/234) in Kenya. Permission and approval were obtained from the National Commission for Science and Technology, the Ministry of Education and principals of the participating schools. Written informed consent was obtained from the parents and assent from the students before completing the questionnaires. The students were assured that their participation was voluntary and a right to withdraw from the study without any consequences. The participants were also assured of confidentiality throughout the study and no names or identifying information were indicated on the questionnaires.

### Procedure

Once permission was obtained, the researcher visited the schools selected to interact with the school heads regarding the research's interests and purpose, and to request their permission for the study. The researcher and four research assistants visited the schools, on different days based on the date and time agreed between the researcher and the school heads. On each day of the school visits, the researcher introduced the research assistants to the participants, prior to explaining the purpose of the study to them, after which the participants willing to participate in the study were issued with consent forms for their parents and assent forms which were signed by the participants. On the day of the data collection, the researcher and research assistants who were well trained prior to the study took the participants who had signed consent and assent forms through a self-administered questionnaire. This questionnaire was in English which is the main language of instruction in secondary schools in Kenya. In addition, the anthropometric measurements such as weight and height were taken based on the WHO stepwise approach for the investigation of NCDs risk factors^15^.

### Data collection instrument

Knowledge about risk factors for NCDs was assessed using a structured questionnaire developed from relevant studies^16^, ^17^. A pilot study was carried out on 30 students in one of the schools before the survey to improve the readability and to validate the content of questionnaire. According to Lorga et al.^16^ the set of questions have acceptable reliability coefficients. The questionnaire comprised of various sections: sociodemographic data of the participants which includes age, gender, year of study and socioeconomic status followed by 10 questions on diabetes, 10 questions on hypertension and five questions on cancer. The participants could obtain a maximum score of 10, 10, and 5 respectively, with a total score of 25 correct answers. The questions were close ended with response choices of “Yes”, “No” and “Don't know”. The bloom's cut off points 80%–100%, 60%–79%, and ≤59% was adapted. The scores for knowledge varied from 0-25 and was classified into three levels; High level as 80-100% (20-25); Moderate level as 60-79% (15-19); Low level as 59% and below (0-14 scores).

### Data analysis

Data was captured and analysed using SPSS version 25. Descriptive statistics were employed to describe the participant's socio-demographic information and knowledge variables which were expressed in frequencies, percentages, means and standard deviations. A value of p < 0.05 was considered statistically significant.

## Results

### Sociodemographic characteristics of the study participants

A total number of 1,310 students were approached in the ten selected schools, and 1,281 respondents consented to participate, yielding a response rate of 97.8%. The mean age of the participants was 16.61 (SD±1.509) years with 704 (55%) female and 577 (45%) males. One third of the participants were in form 3 or grade 11 (33.4%, n=428). Overweight and obese participants were 7.5% (87) and 1% (n=12) respectively.

### Knowledge of non-communicable diseases among the participants

The participants were asked questions related to major non-communicable diseases such as diabetes, hypertension and cancer. The scores were based on 25 questions which ranged between 0 (minimum) and 25 (maximum).

### Diabetes

Diabetes was well known to majority of the participants (81.6%, n=1009). About three quarter (63%, n=733) recognized it as a NCD although 27.9% (n=321) knew that it was not curable. More than half of the participants were more familiar with the risk factors of diabetes and this included obesity (51.2%, n=590), childhood obesity (54.3%, n=647) and diet (65.7%, n=776). Majority of the participants (61.5%, n=735) did not know that family history of diabetes maybe a risk for one to develop the condition while half (51.4%, n=606) did not have the knowledge that age may contribute to getting diabetes especially the elderly. Half of the participants (52.2%, n=604) were aware of the complication caused by diabetes such as slow healing of a wound. However, about 80% were not familiar with the normal levels of blood sugar. Results on the knowledge of diabetes is presented in table 2.

**Table 2:** Knowledge of diabetes among the participants

		Correct answer		Incorrect answer	
Questions		n	%	n	%
1.	Do you know diabetes?	1009	81.6	228	18.4

2.	Diabetes is a communicable disease	733	63.0	430	37.0

3.	Diabetes is a curable disease?	321	27.9	828	72.1

4.	Elderly persons are more susceptible or more likely to have diabetes than young people	574	48.6	606	51.4

5.	If your blood-related relatives or family members have diabetes, are you also at risk of having diabetes?	296	24.7	900	75.3

6.	Obese people are more at risk of diabetes than those who are not obese.	590	51.2	563	48.8

7.	People who regularly eat sweet, fried, and fatty food are at risk of having diabetes.	776	65.7	274	34.3

8.	Obese (overweight) children are at risk of diabetes.	647	54.3	543	45.7

9.	If someone has diabetes (kisukari) and they have a wound (jeraha) it will not heal easy or it might get worse.	604	52.2	554	47.8

10.	Do you know what the normal levels of blood sugar is?	233	20.5	902	79.5

**Note:** Correct answer is *yes

### Hypertension

Almost three quarter (74.2%, n=902) of the study sample were familiar with the term “hypertension”. Stress and strain were well known by the majority (82.9%, n=977) of the participants as risk factors for hypertension. However, lifestyle risk factors of hypertension were not well known, for instance obesity (58.3%, n=669), high salt intake (59.3%, n=681), physical inactivity (63.2%, n=708), smoking (69.8%, n=820) and alcohol consumption (65.6, n=741). In addition, signs and symptoms of hypertension was also not well known (43.1%, n=479). Family history as a risk factor of hypertension (18%, n=213) and normal blood pressure levels 413(35.9%) were the least known by the participants. Results on the knowledge of hypertension is presented in table 3.

**Table 3:** Knowledge of hypertension among the participants

		Correct answer		Incorrect answer	
Questions		n	%	n	%
1.	Have you ever heard of the word “hypertension”?	902	74.2	313	25.8

2.	If your blood-related relatives or family members have high blood pressure, are you also at risk of high blood pressure?	213	18.0	970	82.0

3.	Obese (overweight) people are more at risk of hypertension than those who are not obese.	669	58.3	478	41.7

4.	Smoking increases risk for having high blood pressure.	820	69.8	354	30.2

5.	People with consistent stress and tension are at risk of hypertension.	977	82.9	201	17.1

6.	Consuming salty food increases risk of having hypertension	681	59.3	468	40.7

7.	Alcohol drinkers are at risk of having hypertension.	741	65.6	389	34.4

8.	Do you know what the normal blood pressure is?	413	35.9	738	64.1

9.	Regular exercisers are less likely to have hypertension than those who do not exercise.	708	63.2	412	35.8

10.	If you have high blood pressure you might have the following: headache, dizziness, blurred vision, nausea, vomiting, and weakness of the limbs.	632	56.9	479	43.1

**Note:** Correct answer is *yes

### Cancer

Cancer was known to majority (81.8%, n=960) of the study participants. About 69.4% (n=810) did not know of any family member with cancer. Most of the students identified smoking (88.2%, n=1035) and alcohol (78.3%, n=897) as risk factors for cancer, while half (51.6%, n=553) of them did not know that regular physical activity reduces the risk of getting cancer.

## Discussion

This current study assessed the level of knowledge of NCDs and their risk factors among adolescents in Uasin Gishu County, Kenya. The results revealed that majority (62.1%) of the participants had low levels of knowledge of NCD risk factors. These findings were almost consistent with a similar study in Sri-Lanka 18. On the same note, a study that aimed to assess the level of knowledge of modifiable risk factors for NCDs and its associated factors was conducted in Rwanda among adults living with HIV^19^.The authors concluded that the level of knowledge of NCD modifiable risk factors is poor among adults living with HIV and associated good knowledge with high educational status, normotension and a low CD4 cell count. The researchers recommended comprehensive health education in order to raise awareness of NCDs risk factors. Another study sought to determine the knowledge levels of pregnant women on NCDs, using a similar questionnaire as in the present study. In the same study, knowledge of non-NCDs such as anaemia, diarrhoea and malaria were also assessed; More than three quarter (77.3%) of the women scored more than 60%^17^.

The present study determined the knowledge of the participants regarding selected NCDs and found that majority (81.6%) of the participants knew diabetes. This was consistent with Lorga, et al.^16^ whose questionnaire was adapted for the present study. Another study found 77.5% awareness of diabetes mellitus among high school students in India, with more awareness reported among students in private (93.3%) than government (51.3%) schools^20^ and excellent (93.1%) awareness by both male and female participants^21^. In the current study, majority of the students were familiar with lifestyle risk factors of diabetes such as diet and obesity while some students knew the complications of diabetes. However, majority of the participants lacked knowledge of the levels of blood glucose and did not know that strong indicators for diabetes risk is genetic predisposition and family history as reported by WHO^22^ and Valdez, Yoon, Liu, & Khoury^23^.Therefore, there is a need to educate the students so that those with a family history of diabetes can make healthy lifestyle decisions such as a healthy diet and avoiding unhealthy behaviours such as physical inactivity, alcohol abuse and tobacco use^24^.

Cancer was known to the majority (81.8%) of the study participants, as found in studies by Mane, Maganalli and Nawaz^20^ and Gamage and Jayawardana^18^. This may be due to the increasing cases of cancer reported in the recent years. However, almost three quarter (69.4%) of the participants did not know of any family member with cancer. Therefore, it is necessary for individuals to be aware of the necessary precautions at a younger age, in order to prevent cancer and ensure early diagnosis through regular screening. Although most of the students identified alcohol and smoking as risk factors for cancer, half (51.6%) of them did not have the knowledge that regular physical activity reduces the risk of getting cancer. WHO recommends at least 60 min of moderate to vigorous physical activity for ages 5–17 years old^1^. Hence the need for comprehensive school-based health education on physical activity to motivate students to adopt the practice of regular physical exercises.

Similarly, majority of the students in the present study were familiar with the term hypertension, a result that corroborates with that of Lorga et al.^16^ However, lifestyle risk factors of hypertension such as obesity, high salt intake, physical inactivity, smoking and alcohol consumption were unknown to many students. This finding is unlike the results of a study by Thandar, et al.^17^ who reported high knowledge levels of the risk factors of hypertension among pregnant women. Majority of the participants in this study associated hypertension with consistent stress and strain. Very few students in the present study (18%) recognized the role of family history as a risk factor of hypertension as well as the normal blood pressure (35.9%). Previous studies have reported family history as the least recognized cause of hypertension^16^, ^17^ Therefore, health education on hypertension and other related cardiovascular diseases is necessary for the adolescents.

This study has some limitations. This was a cross-sectional study and issues of reverse causality cannot be ruled out. This study also used a newly developed questionnaire which was pretested for reliability. However comparatively, correct answers might have been easy to choose. Despite such limitations, this study has some strengths. To the best of our knowledge, this is the first study to assess the knowledge on common NCDs among secondary school students in Uasin Gishu County. The findings from this study is applicable in designing health education programs for school going adolescents especially in semi-urban areas.

## Conclusion

The general awareness about NCDs and its risk factors in this study population is low. It is important for adolescents to understand NCDs and their risk factors in order to prevent them from establishing unhealthy behaviours and risky lifestyles. Therefore, there is a need to provide broader and more comprehensive school health education on NCDs and their risk factors in secondary schools. In addition, it is critical to implement programmes to improve the practical application of the existing knowledge on NCD prevention by making a strong impact on students' attitudes and practices.

## Figures and Tables

**Table 1:** Distribution of level of Knowledge (n=1281)

Knowledge Level (Score)	Number (n)	Percentage (%)
Mean score 12.7(SD±4.84)		
Low level (0-14)	796	62.1
Moderate level (15-20)	448	35.0
High level (20-25)	37	2.9

**Table 4:** Knowledge of cancer among the participants

Questions	Correct Answer		Incorrect Answer	
	n	%	n	%
Do you know what cancer is?	960	81.8	214	18.2
Do you know anyone in your family with cancer?	295	25.3	810	74.7
Tobacco/cigarettes increases the risk of cancer	1035	88.2	139	11.8
Alcohol increases the risk of cancer	897	78.3	249	21.7
Regular physical activity reduces the risk of getting cancer	553	48.4	591	51.6

**Note:** Correct answer is yes*
